# Seasonal changes of mineral nutrient absorption and allocation in the branch and leaf of *Zanthoxylum bungeanum* ‘Hanyuan’ during the fruit development

**DOI:** 10.3389/fpls.2024.1484762

**Published:** 2024-11-15

**Authors:** Shuaijie Lu, Jing Xv, Yuanjia Gong, Wei Gong, Wenkai Hui, Jing Qiu, Yafang Zhai, Jingyan Wang

**Affiliations:** ^1^ Key Laboratory of National Forestry Administration on Forest Resources Conservation and Ecological Safety in the Upper Reaches of the Yangtze River, College of Forestry, Sichuan Agricultural University, Chengdu, China; ^2^ College of Agronomy and Biotechnology, Southwest University, Chongqing, China

**Keywords:** Chinese prickly ash, leaf, branch, nutrient dynamics, nutrient diagnosis

## Abstract

**Introduction:**

The dynamic changes of mineral nutrients in the leaf and branch of *Zanthoxylum bungeanum* ‘Hanyuan’ during fruit development can serve as a basis for nutrient diagnosis and scientific fertilization.

**Methods:**

The content of Nitrogen (N), phosphorus (P), potassium (K), calcium (Ca), magnesium (Mg), iron (Fe), manganese (Mn), copper (Cu), and zinc (Zn) in the branch and leaf were measured using current-year shoots of 10-year-old *Z. bungeanum* ‘Hanyuan’ during the fruit development period, and the corresponding nutrient content in soil of the orchard were also determined to explore the nutrient demand patterns of *Z. bungeanum* ‘Hanyuan’ trees.

**Results:**

Both branch and leaf exhibited relatively high levels of various nutrients during the early stages of fruit growth, then declined temporarily. At fruit maturity, the content of Ca and K in branches was the highest, while the content of Ca and N in leaves was the highest. At fruit maturity, the average nutrient content of N, P, K, Ca, Mg, Fe, Mn, Cu, and Zn in the branches and leaves were 17.25 g/kg, 1.99 g/kg, 18.84 g/kg, 26.14 g/kg, 3.69 g/kg, 215.61 mg/kg, 248.85 mg/kg, 13.08 mg/kg, and 53.77 mg/kg. The N, K, Ca, Fe, Cu, and Zn content in the branches and leaves significantly correlated with those nutrients content in the soil.

**Discussion:**

The appropriate period for nutrient diagnosis of *Z. bungeanum* is 39−86 d after flowering (AF), with the critical period for branch and leaf nutrient requirements being 1−39 d AF. This provides a basis for nutrient supplements in *Z. bungeanum* ‘Hanyuan’ orchards management.

## Introduction

*Zanthoxylum bungeanum* is a deciduous tree species of the Rutaceae family and *Zanthoxylum* genus. The fruit of Chinese prickly ash is rich in volatile oils (Huang et al., 2022; Fei et al., 2020) and amide compounds (Zhang et al., 2023), which contribute to their distinctive flavor profile. These qualities make them indispensable in Chinese culinary culture, particularly in Sichuan cuisine, where they hold a pivotal role. Except for fruit, the leaf of Chinese prickly ash also contains oil sacs that contribute to the distinctive aroma. Moreover, they also contain a variety of secondary metabolites with significant potential for consumption (Zhang et al., 2014), and antimicrobial, and antioxidant properties (Ma et al., 2022). The reproduction of Chinese prickly ash is divided into sexual and asexual propagation (Fei et al., 2021), with softwood cuttings being the primary means of asexual reproduction (Singh and Rawat, 2016). In addition, Chinese prickly ash tree sprouts, including young shoots, leaves, and stems, are highly popular in China due to their rich nutritional content and distinctive flavor (Wang et al., 2023). Chinese prickly ash holds significant economic value, with China being its primary producer (Chen et al., 2022; Feng et al., 2016) and the largest exporter in the world. *Z. bungeanum* ‘Hanyuan’ is one of the optimal cultivars in Sichuan Province, China, famous for its excellent quality. It is extensively cultivated in Hanyuan County and surrounding areas, with a planting area exceeding 13300 hm^2^. By the end of 2023, the brand value assessment of *Z. bungeanum* ‘Hanyuan’ reached 6.104 billion RMB, indicating it has broad prospects for development. The fertilization measures and quantity are usually unscientific and irrational and result in abnormal plant growth, fertilizer waste, and environmental pollution in the management of Chinese prickly ash (Zhao et al., 2021). These cultivation management problems also have arisen with the increase of planting area of *Z. bungeanum* ‘Hanyuan’ and need to be solved to ensure the sustainable development of *Z. bungeanum* ‘Hanyuan’.

Determining the content of mineral nutrients in leaves at different developmental stages is an effective method for assessing plant nutritional status and guiding fertilization practices (Uçgun and Gezgin, 2017). The period for leaf nutrient diagnosis varies among different species. Stateras and Moustakas (2017) formulated optimal fertilization formulas and scheduling for olive orchards by studying nutrient fluctuation characteristics in olive leaves across different seasons. Oliosi et al. (2020) assessed the nutritional status of various nutrients based on the changes in nutrient contents in coffee leaves and proposed corresponding fertilization recommendations. Apart from leaves, branches and stems can also serve as organs for assessing plant nutrient status. Marathe et al. (2016) studied nutrient contents in the leaf, branch, and root of pomegranate, finding that nutrient content in the stem was more stable than that in the leaf. Golomb and Goldschmidt (1987) discovered that the nutritional composition of citrus twigs is influenced by fruit development. Similarly, Mirsoleimani et al. (2014) found that certain nutrient contents in branches significantly affect the alternate bearing phenomenon of citrus trees. Studies on assessing tree nutrient status based on plant nutritional dynamics have also been reported for fig (Benou et al., 2020), orange trees (Dias et al., 2013), and avocado (Salazar-García et al., 2019). Some scholars argued that the period for nutrient diagnosis should be during dormancy (Schreiner, 2020), while others suggested it should coincide with the peak of plant growth (Dias et al., 2017). However, whether during dormancy or active growth stages, it is generally agreed that various metabolic activities of the tree should remain relatively stable during nutrient diagnosis (Salazar-García et al., 2018). For Chinese prickly ash, all leaves fall off every autumn, and new leaves sprout from March to April in the following year, nutrient diagnosis should be conducted during the developmental period. Understanding the growth and development patterns of *Z. bungeanum* ‘Hanyuan’ branch and leaf, and revealing its nutrient demand characteristics, provides a scientific basis for targeted optimization of fertilization management, which has great significance for the green and sustainable development of the *Z. bungeanum* ‘Hanyuan’.

Currently, there are fewer studies related to the growth and nutrient dynamics of Chinese prickly ash. In previous research, we researched the changes in nutrient content and accumulation in the fruits of *Z. bungeanum* ‘Hanyuan’ (Lu et al., 2024). By sampling and investigating the new branch and leaf of 10-year-old *Z. bungeanum* ‘Hanyuan’ during different growth periods, the objectives of this study were to (1) combine previous research on fruit parts, comprehensively evaluate the nutrient absorption patterns and accumulation of nitrogen (N), phosphorus (P), potassium (K), calcium (Ca), magnesium (Mg), iron (Fe), manganese (Mn), copper (Cu), and zinc (Zn) in the current-year organs of *Z. bungeanum* ‘Hanyuan’, (2) and determine the period when the tree nutrients are relatively stable, provides a scientific basis for the nutrient diagnosis of *Z. bungeanum* and nutrient evaluation of Chinese prickly ash orchards. This study can provide a basis for the timing and quantitative fertilization of Chinese prickly ash orchards during different growth periods, as well as offer a theoretical foundation for improving fertilization management practices in the Chinese prickly ash industry in Sichuan, contributing to the green and sustainable development of the industry.

## Materials and methods

### Plant materials and experimental design

**Figure 1:**
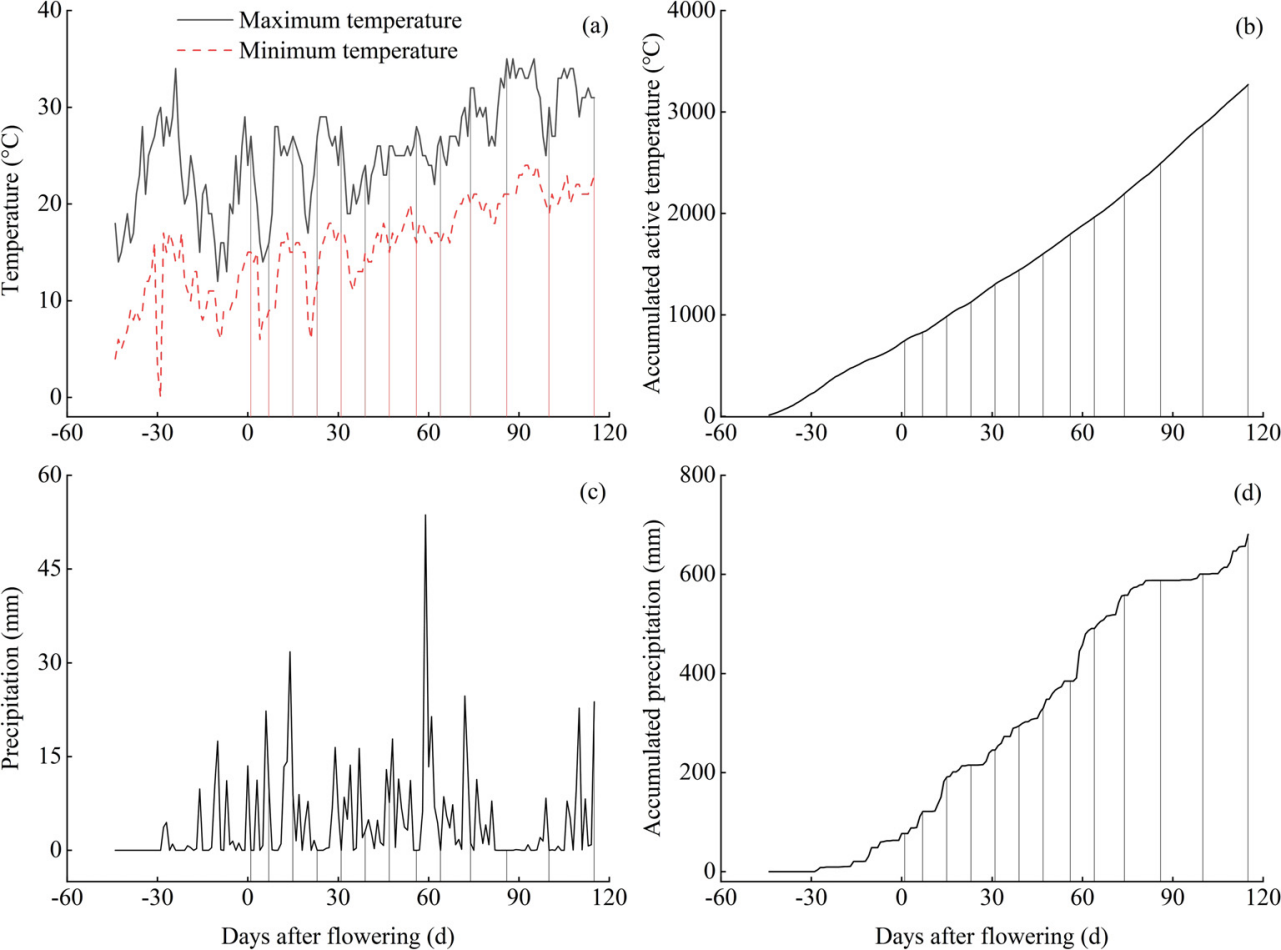
Daily variations and cumulative amounts of temperature and precipitation during the growing period. The vertical lines in the figure correspond to different sampling times.

The experimental site is located in Hanyuan County, Ya’an City, Sichuan Province, China (102°37′ E, 29°39′ N), and belongs to a traditional cultivation area for Chinese prickly ash. The area is a typical subtropical monsoon humid climate, with an annual average air temperature of 17.9°C, frost-free period 300 d, sunshine hours of 1475.8 h, active accumulative temperature of 5844.7°C, and an average annual rainfall of 741.8 mm. The soil type is yellow loam with the following initial properties: pH 5.84, organic matter of 37.78 g kg^−1^, total N 1.62 g kg^−1^, total P 1.46 g kg^−1^, and total K 14.27 g kg^−1^. The plant materials tested in this study were 10-year-old Chinese prickly ash (*Zanthoxylum bungeanum*) cultivar of ‘Hanyuan’, and the planting space was 2 m × 3 m. The changes in temperature and precipitation at the experimental site during the sampling period in 2022 are shown in **Figure 1**. The starting date for temperature and precipitation statistics is from when the temperature exceeded the biological minimum temperature for Chinese prickly ash, until the end of sampling. In this study, the biological minimum temperature for Chinese prickly ash is selected as 10°C (Zheng et al., 2021).

The fertilizers were applied in late February (100 g of urea, 200 g of superphosphate, and 100 g of potassium sulfate per tree), early May (100 g of urea, 200 g of superphosphate, and 100 g of potassium sulfate per tree), and late August (250 g of urea, 500 g of superphosphate, and 250 g of potassium sulfate per tree) in the *Z bungeanum* ‘Hanyuan’ orchard. Fertilization and irrigation were carried out simultaneously. The tricyclazole, mancozeb, and imidacloprid were used to control pests and diseases during the fruit development stage.

The experiment was conducted as a field trial using a completely randomized design. Three plots (10 m × 10 m) with the same soil type, similar elevation (1740 m), aspect (SW 223°, and slope (7°) were selected. Five representative *Z. bungeanum* ‘Hanyuan’ trees with similar growth were selected for sampling in each plot, and another three representative *Z. bungeanum* ‘Hanyuan’ trees with similar growth were selected and unsampled for biomass estimation. The branches and leaves were sampled from the selected trees every 1–2 weeks after flowering (AF). The branches and leaves sampling time was recorded as 1 d AF and continued to harvest (115 d AF) at the mature stage. The specific sampling times included 1 d, 7 d, 15 d, 23 d, 31 d, 39 d, 47 d, 56 d, 64 d, 74 d, 86 d, 100 d, and 115 d AF.

At each sampling stage, 1–3 representative branches were collected from each of the four directions of the 5 sample trees in every plot, and a total of 20–60 new branches were sampled. The branches and leaves were separated and placed into the portable freezers, and then immediately transported to the laboratory. The surface of the branches and leaves was cleaned with deionized water. All samples were dried to constant weight in an oven at 65°C, and used for relevant parameters determination.

The soil samples in 0−40 cm layer were collected at the same time as branches and leaves due to the Chinese prickly ash tree being a shallow-rooted species (Liao et al., 2016), five sampling points were selected in each plot. The samples at the same soil layer in each plot were thoroughly mixed, and approximately 1 kg of soil was selected using the quartering method. These soil samples were then brought back to the laboratory, naturally air-dried, and ground for determination of nutrient content.

### Nutrient content determination

The dried plant samples were ground and passed through a 0.25-mm sieve and digested by using concentrated nitric acid (HNO_3_) and perchloric acid (HClO_4_) (Jashnsaz and Nekouei, 2016). The K, Ca, Mg, Fe, Mn, Cu, and Zn content were measured by inductively coupled plasma-Mass Spectrometry (ICP-MS, NexION1000G) (Jo and Todorov, 2019). The P content was measured by the molybdenum antimony anti-colorimetric method (Singh et al., 2020), and the N content was measured by the Kjeldahl determination method (Czubinski et al., 2021).

The air-dried soil samples were ground and passed through a 2-mm sieve. The alkali diffusion method was used to determine the alkali-hydrolysable N content in soil, while the available P was extracted with sodium bicarbonate (NaHCO_3_) and measured using the molybdenum antimony anti-colorimetric method. The available K and exchangeable Ca and Mg were extracted with ammonium acetate (NH_4_AOC), while the available Fe, Mn, Cu, and Zn were extracted with diethylenetriaminepentaacetic acid (DTPA) and measured using ICP-MS (Nazli and Erdal, 2022).

### Statistical analysis

The total biomass of leaves and branches was calculated by multiplying the average biomass during a specific period by the total number of that organ during that period. Nutrient accumulation in leaves and branches was determined by the average nutrient content multiplied by the average dry weight. Excel 2010 and SPSS Statistics 27 software were used for statistical analysis, and Graph Pad software was used for drawing. Indices at different development stages on the content and accumulation of leaf and branch were analyzed by one-way ANOVA, followed by a *post-hoc* test that employed Duncan’s multiple range test to compare the differences in nutrient content and accumulation between different periods. Pearson correlation analysis was used to estimate the relationship between plant (including leaves and branches) nutrient content and soil available nutrient content. In addition, the total nutrient accumulation in the current-year organs of *Z. bungeanum* ‘Hanyuan’ included branches, leaves, and fruits, and the data on nutrient accumulation in fruits has been reported in previous research (Lu et al., 2024).

## Results

### Branch and leaf growth

**Figure 2:**
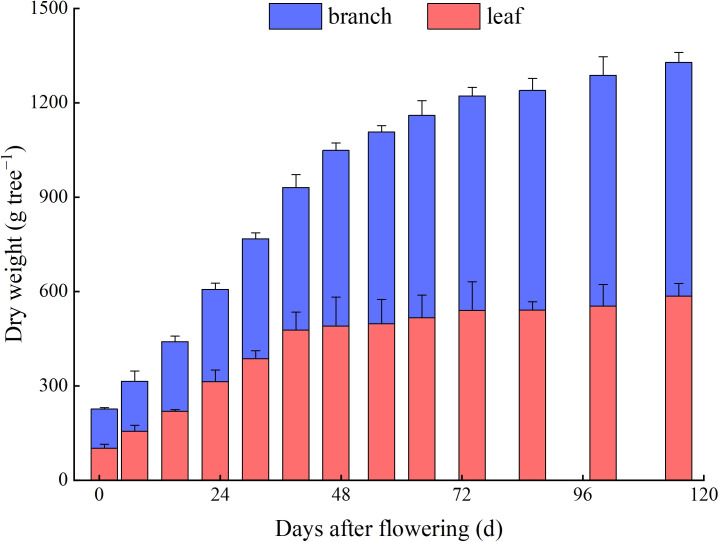
Dynamic changes of the branches and leaves dry weight in individual trees.

The biomasses of branches and leaves in *Z. bungeanum* ‘Hanyuan’ plants were increased continuously during their development. The lowest values were observed at 1 d AF, with 124.9 g per plant for branches and 101.6 g per plant for leaves. These highest values were observed at 115 d AF, with 742.5 g per plant for branches and 584.4 g per plant for leaves. The branches dry weight had the fastest increase at 1−47 d AF, reaching 558.3 g per plant at 47 d AF, which accounted for 75.2% of the biomass at the fruit maturity. The leaves dry weight had the fastest increase at 1−39 d AF, reaching 477.2 g per plant at 39 d AF, which accounted for 81.5% of the biomass at the fruit maturity (**Figure 2**).

### Nutrient contents in branches and leaves

The content of N, P, Mg, and Mn were higher in the leaves than in the branches during the fruit development periods. The content of K and Ca were higher in the branches than in the leaves before 47 d AF, but both showed a higher level in the leaves after 47 d AF. The content of Fe, Cu, and Zn in the branches and leaves fluctuated continuously throughout the entire development period.

**Figure 3:**
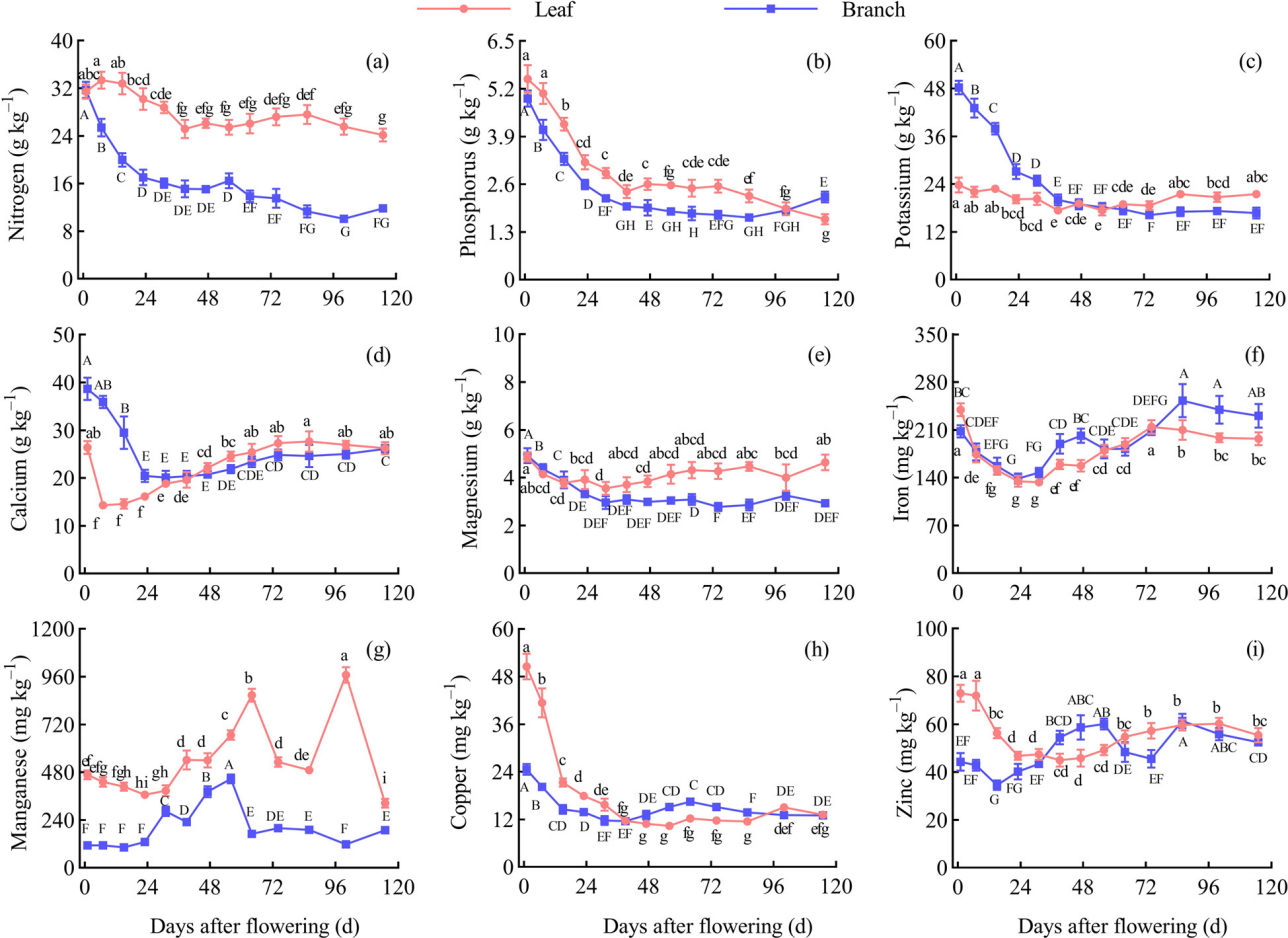
Dynamic changes of the nutrient contents in the leaves and branches. Different capital letters and lowercase letters denote significant differences at the *P* < 0.05 level for the branches and leaves, respectively, at the different stages. **(A)** Nitrogen content. **(B)** Phosphorus content. **(C)** Potassium content. **(D)** Calcium content. **(E)** Magnesium content. **(F)** Iron content. **(G)** Manganese content. **(H)** Copper content. **(I)** Zinc content.

In the leaves, the content of P, K, Mg, Fe, Cu, and Zn reached peak values were 5.47 g/kg, 23.79 g/kg, 4.86 g/kg, 239.40 mg/kg, 50.57 mg/kg, and 73.00 mg/kg, respectively, at 1 d AF. The content of N reached the peak value was 33.34 g/kg at 7 d AF, while the content of Ca and Mn reached peak values were 27.67 g/kg and 968.60 mg/kg at 86 d AF and 100 d AF, respectively. Throughout the entire growth periods, the N and P content in leaves overall showed a decreasing trend; the K and Mg content remained relatively stable; the Ca, Fe, Cu, and Zn content showed an initial decline followed by an overall increased trend; the Mn content exhibited two peaks at 64 d AF and 100 d AF, respectively. Overall, the N, P, Ca, Fe, Cu, and Zn in the leaves exhibited a rapid decline during the early development stage, with significant differences between adjacent periods (*P* < 0.05). Except for Fe and Mn, the other elements remained relatively stable at 39−86 d AF, with no significant differences between adjacent periods (*P* > 0.05) (**Figure 3**).

In the branches, the content of N, P, K, Ca, Mg, and Cu reached peak values were 31.73 g/kg, 4.94 g/kg, 48.27 g/kg, 38.66 g/kg, 4.92 g/kg, and 24.63 mg/kg, respectively, at 1 d AF. The content of Mn reached peak value was 446.08 mg/kg at 56 d AF, and the content of Fe and Zn reached peak values were 253.04 mg/kg and 61.44 mg/kg, respectively, at 86 d AF. Throughout the entire growth periods, the content of N, P, K, and Mg in branches demonstrated a continuous declined trend; the content of Ca showed an initial decline followed by a continuously increased trend; the content of Fe, Cu, and Zn fluctuated; the content of Mn showed a peak of increased at 56 d AF. Overall, the N, P, K, Ca, Mg, Fe, and Cu in the branches exhibited a rapid decline during the early development stage, with significant differences between adjacent periods (*P* < 0.05). P and Mg content remained stable at 39−86 d AF, while K, Ca, Mn, and Cu content stabilized at 64−100 d AF, showing no significant differences between adjacent periods (*P* > 0.05) (**Figure 3**).

**Figure 4:**
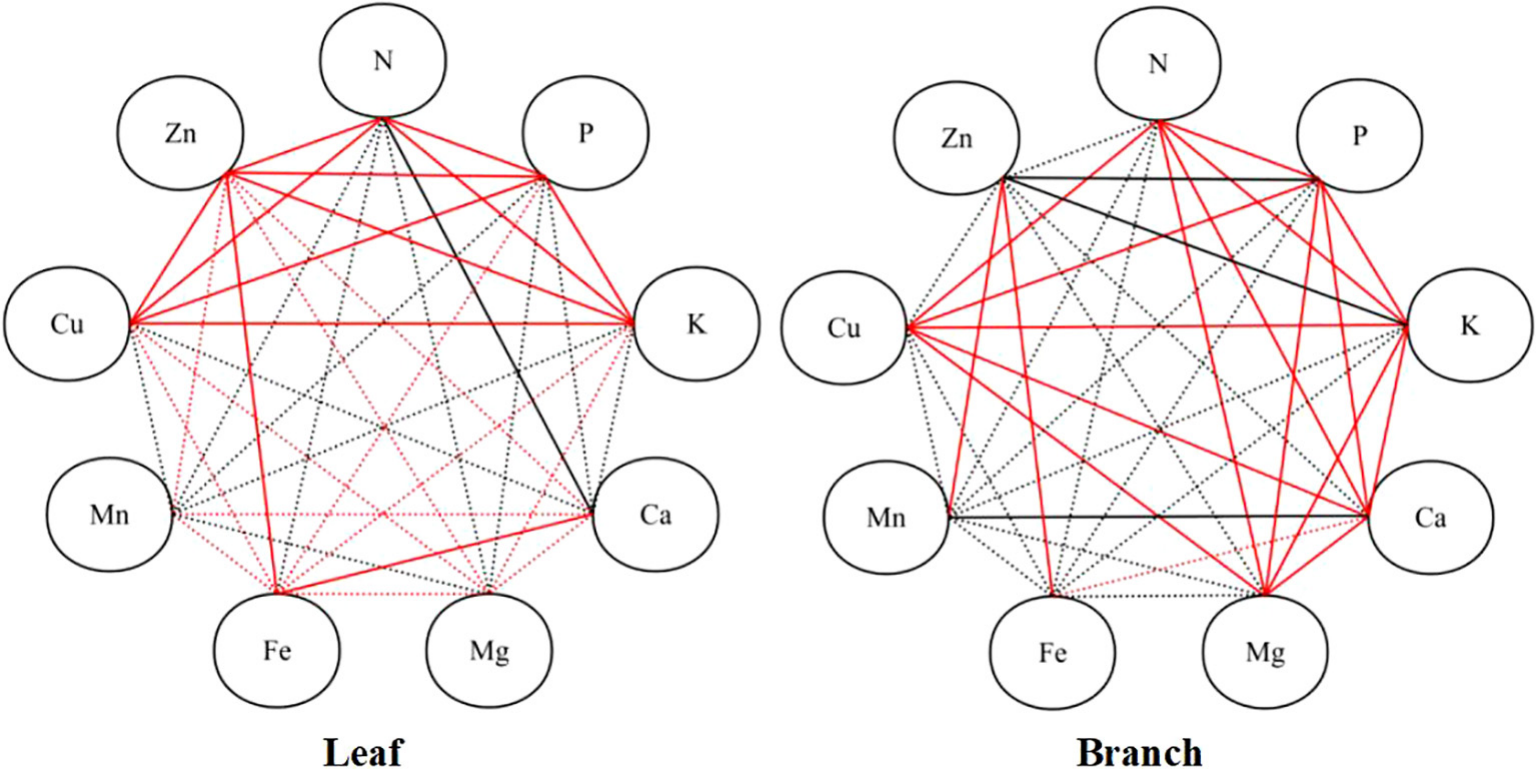
Pearson correlation analysis of nutrient contents in the branches and leaves. Red solid lines refer to significantly positive correlations among the elements (*P* < 0.05), black solid lines refer to significant negative correlations among the elements, red dotted lines refer to positive correlations among the elements, and black dotted lines refer to negative correlations among the elements.

In the leaves, the N content with P, K, and Cu content, the P content with K, Cu, and Zn content, the K content with Cu, and Zn content, the Ca content with Mg, and Fe content, the Mg content with Fe, and Zn content, and the Zn with Fe, and Cu content were observed significantly and positively correlated (*P* < 0.05); while the significant negative correlation was observed between N and Ca content (*P* < 0.05). In the branches, significant positive correlations were observed among the N, P, K, Ca, and Mg content, as well as the Cu content with N, P, K, Ca, and Mg content, the Zn content with Fe, and Mn content; while the Mn content with Ca content, and the Zn content with P, and K content were observed significantly and negatively correlate (*P* < 0.05) (**Figure 4**).

### Nutrient accumulation in branches and leaves

**Figure 5:**
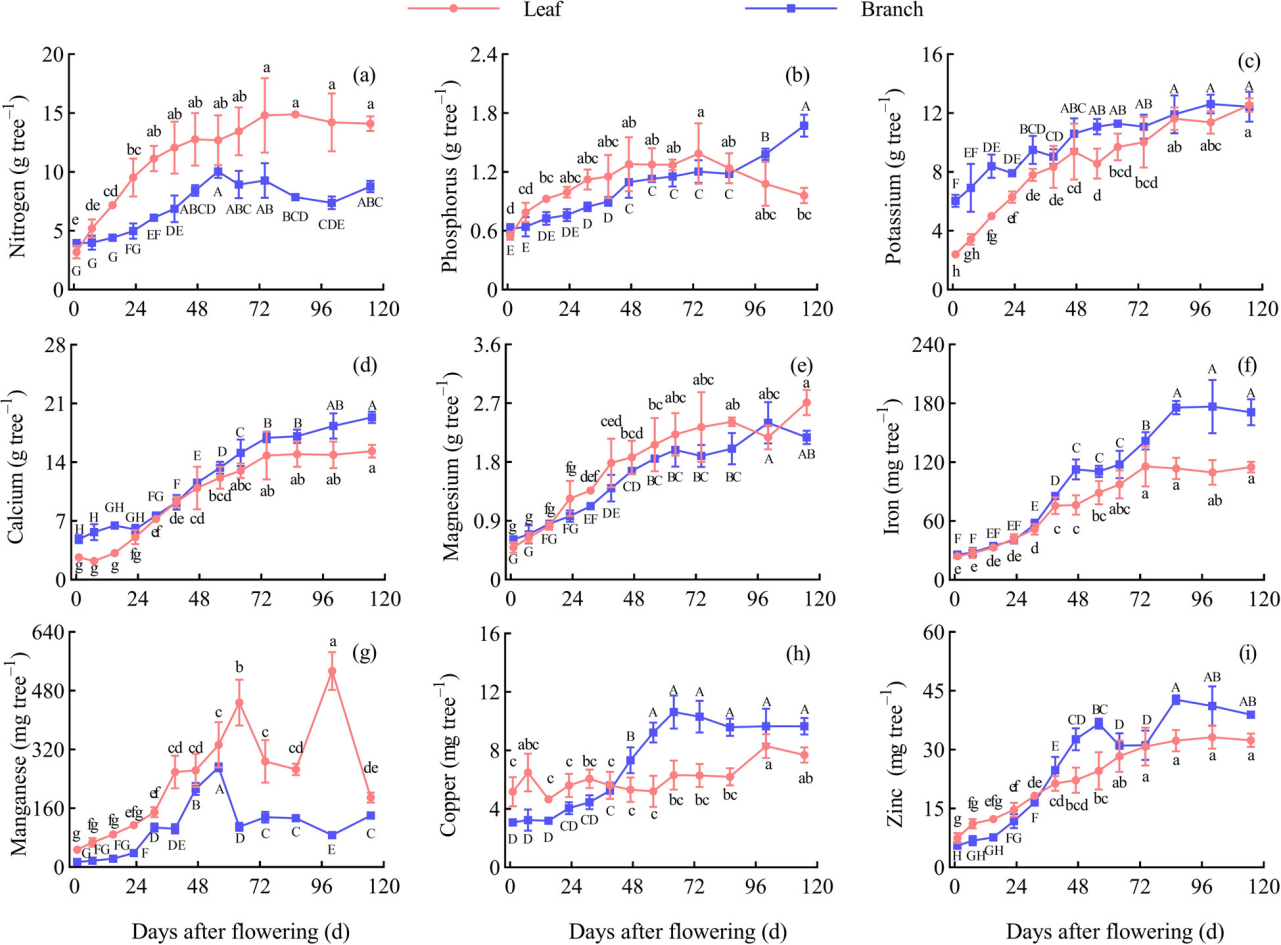
Dynamic changes of the nutrient accumulation in the leaves and branches. Different capital letters and lowercase letters denote significant differences at the *P* < 0.05 level for the branches and leaves, respectively, at the different stages. **(A)** Nitrogen content. **(B)** Phosphorus content. **(C)** Potassium content. **(D)** Calcium content. **(E)** Magnesium content. **(F)** Iron content. **(G)** Manganese content. **(H)** Copper content. **(I)** Zinc content.

The nutrient accumulation overall showed an increasing trend during the entire development period, with a relatively rapid growth in the early stages and a slower growth in the later stages. The accumulation of N, K, Mg, and Mn during the maturity period was higher in the leaves than in the branches, while the accumulation of the other nutrients was higher in the branches (**Figure 5**).

In the leaves, except for Cu, the accumulation of the other nutrients was the fastest before 39 d AF, with significant differences observed between adjacent periods (*P* < 0.05). and the accumulation increased slowly at 39−74 d AF. The accumulation of N and P began to decline after 74 d AF, but the Ca, Mg, Fe, and Zn accumulation remained stable. The Cu remained relatively stable during the entire growth period, and the Mn showed two peaks (**Figure 5**).

The nutrient accumulation showed a different change pattern in the branch. The N accumulation continuously increased before 56 d AF and began to decline after 56 d AF. The P accumulation remained stable at 47−86 d AF and increased during the other periods. The K, Ca, Mg, and Fe accumulation overall showed an increasing trend. The Mn accumulation showed a peak at 56 d AF. The Cu accumulation continuously increased before 64 d AF and then decreased. The Zn accumulation decreased at 56−74 d AF and 86−115 d AF and increased during the other periods (**Figure 5**).

The accumulation of N, P, K, Ca, Mg, Fe, Mn, Cu, and Zn in branches and leaves of a *Z. bungeanum* ‘Hanyuan’ tree at fruit maturity were 22.89 g, 2.64 g, 25.00 g, 34.69 g, 4.89 g, 286.11 mg, 330.22 mg, 17.36 mg, and 71.35 mg. The average nutrient content in the branches and leaves were 17.25 g/kg, 1.99 g/kg, 18.84 g/kg, 26.14 g/kg, 3.69 g/kg, 215.61 mg/kg, 248.85 mg/kg, 13.08 mg/kg, and 53.77 mg/kg, respectively. The order of nutrient accumulation in the branches during the maturity stage (115 d AF) was Ca > K > N > Mg > P > Fe > Mn > Zn > Cu, while in the leaves the order was Ca > N > K > Mg > P > Mn > Fe > Zn > Cu.

### The variation of available and exchangeable nutrient contents in soil

**Figure 6:**
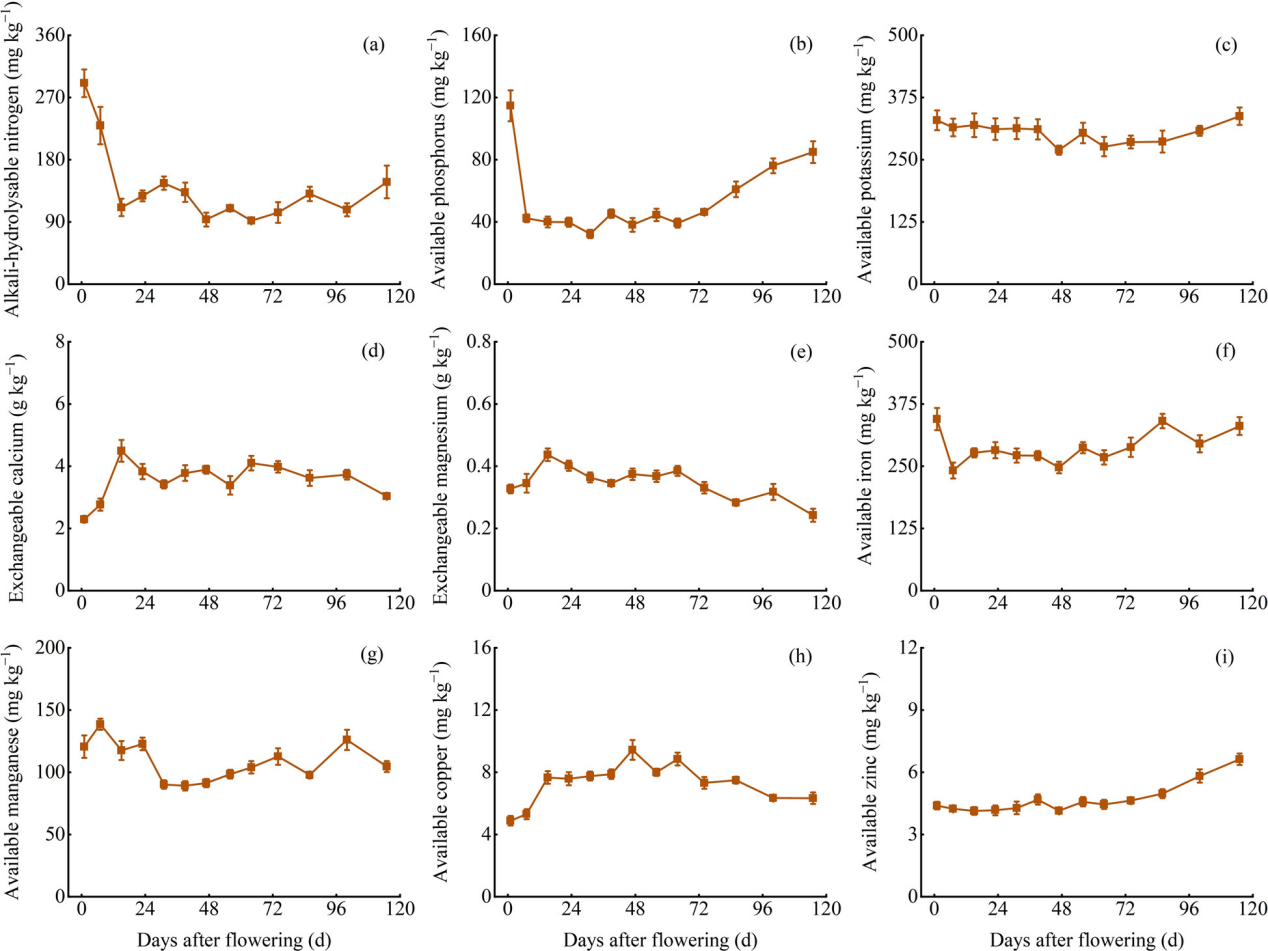
Dynamic changes of alkali-hydrolysable N, available P, K, Fe, Mn, Cu, and Zn, exchangeable Ca, and Mg content. **(A)** Alkali-hydrolysable nitrogen content **(B)** Available phosphorus content. **(C)** Available potassium content. **(D)** Exchangeable calcium content. **(E)** Exchangeable magnesium content. **(F)** Available iron content. **(G)** Available manganese content. **(H)** Available copper content. **(I)** Available zinc content.

The content of alkali-hydrolysable N, available P, available K, exchangeable Ca, exchangeable Mg, available Fe, available Mn, available Cu, and available Zn in soil during the branch and leaf development were 91.90−290.75 mg kg^−1^, 32.28−114.68 mg kg^−1^, 269.34−337.39 mg kg^−1^, 2.30−4.49 g kg^−1^, 0.24−0.44 g kg^−1^, 241.45−344.43 mg kg^−1^, 89.12−152.31 mg kg^−1^, 4.88−9.43 mg kg^−1^, and 4.13−6.62 mg kg^−1^, respectively. Generally, available K content remained stable, while exchangeable Ca, exchangeable Mg, and available Cu content initially increased and then decreased, while the other nutrient contents showed an initial decrease and then a continuous increase. Furthermore, the content of alkali-hydrolysable N, available K, exchangeable Ca, available Fe, available Cu, and available Zn in soil was significantly correlated (*P* < 0.05) with the content of N, K, Ca, Fe, Cu, and Zn in *Z. bungeanum* ‘Hanyuan’ branches and leaves (**Figure 6**).

## Discussion

The nutrient requirement of current-year organs (mainly including fruits, branches, and leaves) is important to make effective fertilization measures (Moustakas et al., 2011). Based on the previous and present research that focused on the nutrient demands of *Z. bungeanum* ‘Hanyuan’ fruits (Lu et al., 2024) and current-year shoots (including branch and leaf), our findings revealed that the N, P, K, Ca, Mg, Fe, Mn, Cu, and Zn absorption of current-year organs (including branches, leaves, and fruits) were 88.15 kg/ha, 11.05 kg/ha, 103.87 kg/ha, 129.37 kg/ha, 17.25 kg/ha, 1.32 kg/ha, 0.93 kg/ha, 64.57 g/ha, and 272.03 g/ha, respectively, at the mature stage. Roccuzzo et al. (2012) provided the basis for the fertilization amount of orchards by assessing the dynamic nutrient absorption of different organs of oranges during the growth stages. Similar studies have also been reported in grapes (Conradie et al., 2022) and citrus (Liu et al., 2020). The nutrient demand of branches and leaves of *Z. bungeanum* ‘Hanyuan’ is primarily concentrated during the 1−39 d AF. Significant differences (*P* < 0.05) exist between different periods during this stage, and the nutrient accumulation in branches and leaves has already exceeded 60% of the amount absorbed during the mature stage. During this time, branches and leaves grow rapidly, leading to a rapid increase in nutrient accumulation. Similar changes have also occurred in tobacco, where the nutrient absorption can reach more than 80% of that at the mature stage when it grows for 35 days (Moustakas and Ntzanis, 2005). Similar changes have also been reported in oranges fruits (Roccuzzo et al., 2012). Appropriate fertilization measures can enhance plant nutrient status. The fertilization period can be selected based on the nutrient accumulation amount of trees at different growth stages. Based on our study results, we recommend that *Z. bungeanum* ‘Hanyuan’ should receive fertilizers application within one month after flowering, primarily using N, P, K, and Ca fertilizers, supplemented with Mg and micronutrients. Additionally, it is important to consider fertilizer types, soil nutrient status, and fertilizer utilization efficiency. This study can provide a reference for the timing and quantitative fertilization of Chinese prickly ash orchards.

Nutrient diagnosis in Chinese prickly ash trees is beneficial for growers to assess the nutritional status and adjust fertilization practices to enhance yield and quality (Cavalcanti et al., 2021). Currently, leaf analysis is a primary method because the leaf is a storage and transport organ for nutrients and is easily sampled and analyzed (Matsuoka, 2020). Except for leaf, other plant organs such as branches (Johnson et al., 2006), flowers (Wójcik and Filipczak, 2019; Khelil et al., 2010), and wood (Clark et al., 1986) have also been reported for nutrient diagnosis. The content of various nutrients in tree organs should be stable during nutrient diagnosis (Salazar-García et al., 2018), as dynamic changes can significantly interfere with accurate assessment (Walworth and Sumner, 1987). Therefore, identifying suitable nutrient diagnosis organs and periods during plant development is important. In this study, the N, P, K, Mg, Cu, and Zn content in the leaves was found to remain stable at 39−86 d AF, while the Fe content in the leaves remained stable at 86−115 d AF; In the branch, the P and Mg content remained stable at 39−86 d AF, and the K, Ca, Mn, and Cu content remained stable at 64−100 d AF. There were no significant differences between the periods within this time range (*P* > 0.05), thus making it suitable for nutrient diagnosis in the respective organs. Tree nutrient diagnosis should consider the content and change of nutrients in different organs, and besides leaves, other tree organs also can provide valuable information. Furthermore, integrating plant organs with soil for nutrient analysis can provide a better understanding of plant nutrition status and nutrient management (Sema et al., 2010). The N, K, Ca, Fe, Cu, and Zn content in soil significantly correlated with those nutrients content in current-year shoots (including branches and leaves) during the different stages in the present study. This result implies the significant impact of soil nutrient status on plant nutrient content, soil nutrient status is also a necessary consideration when evaluating the nutrient status of *Z. bungeanum*. This study provides a reference for the nutritional assessment of *Z. bungeanum* trees and the orchard soil, identifying suitable periods for nutritional diagnosis using branches and leaves. Based on the data and trends from this research, growers can accurately assess the nutritional status of *Z. bungeanum*, laying the groundwork for smart agriculture and helping the industry adopt more sustainable fertilization practices. However, further research is needed to systematically study nutrient variations across various organs under different soil conditions.

The nutrient content in soil significantly influences tree growth and other physiological processes (Heineman et al., 2021). Different plant species have different nutrient requirements, and soil nutrient management is important to ensure robust plant growth. The content of alkali N, available P, available Fe, and available Mn in soil showed high levels before flowering stage, possibly due to lower nutrient demands during winter dormancy (Kaštovská et al., 2022), leading to an increase in soil nutrient content during this period. Dong et al. (2001) found that apple trees absorb fewer nutrients from winter to early spring, when soil temperature is low and above-ground growth is inactive, suggesting that fertilization may not be necessary during this time. During the flowering period of *Z. bungeanum ‘*Hanyuan’, some nutrient contents declined rapidly, which may be related to increased tree demand, thus timely supplementation of soil nutrients is important to promote organ development. Apart from this initial decline, several available nutrients in soil showed a slight decrease at 56 d AF in the present research, possibly due to high nutrient demands and absorption during tree growth and fruit development. *Z. bungeanum* ‘Hanyuan’ fruit development generally follows the double sigmoid curve, indicating a critical period of increased nutrient demand at 56 d AF (Lu et al., 2024). Aira et al. (2021) observed significant declines in various soil nutrient contents during the growth of fern species. In this study, the available soil nutrients overall showed an increasing trend, it was possible due to rising temperatures following the branch and leaf development, which not only promotes *Z. bungeanum* ‘Hanyuan’ tree and root growth but also accelerates organic matter input into the soil (Yang et al., 2016). This enhances soil microbial growth and activity (Min et al., 2019; Gomez et al., 2020), which facilitates the conversion of soil nutrients and increases available nutrient contents. A similar phenomenon has been reported in nutrient dynamics in subalpine forests (Tan et al., 2011). Therefore, appropriate fertilization should be applied during the early growth stages of *Z. bungeanum* ‘Hanyuan’ and at 56 d AF. During the early growth stages, the soil should predominantly be supplemented with N, P, and K fertilizers. At 56 d AF, all nutrients can be supplemented, but the amount should be relatively smaller to maintain an adequate supply of soil nutrients.

The content of various nutrients showed a rapid decreasing trend during the early growth stages of the branch and leaf, with significant differences observed in nutrient content between adjacent periods (*P* < 0.05). The decline in nutrient content is mainly due to a large amount of dry matter produced by leaf photosynthesis and allocated to the various organs of the tree during this period (Liu et al., 2020), and the rate of dry matter accumulation is higher than that of nutrient absorption, then resulting in a ‘dilution effect’ for nutrients. Waterland et al. (2017) observed a decline in nutrient content during rapid leaf growth in Kale. Similarly, *Zanthoxylum armatum* ‘novemfolius’ leaf also showed comparable patterns (Wang et al., 2018). The nutrient content decline in the branches and leaves during early stages may be influenced not only by their development but also by the fruiting process. The leaf is the main nutrient source during fruit growth and development (Ali et al., 2016), and fruit nutrient absorption leads to declined nutrient contents in the leaf. Therefore, applying the appropriate amount of additional fertilizer between 1−39 d AF in *Z. bungeanum* ‘Hanyuan’ is crucial to maintaining tree nutrient contents. Additionally, the earlier fertilization before the flowering period may obtain a better effect. The types of fertilizers should be determined based on the changing trends, with N, P, K, and Ca showing the greatest decline and higher content in the branches and leaves, which should be prioritized. This practice will promote the branch, leaf, and fruit development, then increase the fruit yield and quality.

Chinese prickly ash sprouts, including new leaves and shoots, are considered woody vegetables rich in proteins and minerals. The elevated nutrient contents in early growth stages contribute significantly to their popularity (Wang et al., 2023). Therefore, Chinese prickly ash sprouts should be harvested approximately at 15 d AF, when the nutrient content and biomass of branches and leaves are comparatively high to ensure better quality. By determining the optimal harvest time for Chinese prickly ash sprouts, farmers can increase their planting income and enhance the vitality of the Chinese prickly ash industry, contributing to its long-term development.

The functions of nutrients vary across different plant organs, influencing their accumulation and distribution. N, P, and K are essential macronutrients that significantly contribute to plant growth and development, while Ca and Mg are critical for maintaining cellular structure and facilitating photosynthesis. Additionally, micronutrients such as Fe, Mn, Cu, and Zn play pivotal roles in activating various enzymatic processes and redox reactions within plants. Studying nutrient allocation in the leaves and branches contributes to understanding the tree life processes. In this study, the P accumulation in the branches showed a rapid increase at 74 d AF, while the P accumulation in the leaves decreased rapidly. Additionally, the P accumulation in the branches was higher than that in the leaves at fruit maturity. Kong et al. (2023) found that *Z. armatum* ‘novemfolius’ P content in the branches showed an increasing trend approximately 50 days before fruit maturity. This trend may be related to bud formation in autumn when the branch serves as the primary developmental organ for buds (Gou et al., 2017). Higher P content promotes bud formation and growth (Li et al., 2023). Zhou et al. (2020) found that the highest P content (N_2_P_3_K_1_) significantly enhances flower bud differentiation. Furthermore, the Mg content in the leaf was higher than that in the branch, potentially associated with chloroplasts in the leaf. The Mg is crucial for chloroplast function as a central ion in chlorophyll and a cofactor for photosynthetic enzymes. The Mg deficiency can impair photosynthesis and chloroplast formation, affecting overall plant growth and development (Dukic et al., 2023). Nutrient content variations stem from metabolic activities, physiological functions, and management practices. In the management of Chinese prickly ash orchards, scientific nutrient management should be strengthened according to the physiological activities and nutrient requirements of different organs to satisfy the nutrient requirements of the plants during the fruiting stage.

Mancozeb, a non-systemic dithiocarbamate fungicide, effectively controls various plant diseases such as downy mildew, black spot, and rust (Araújo et al., 2020; Carniel et al., 2019), playing a crucial role in the daily management of Chinese prickly ash orchards. This study found that the Mn content in *Z. bungeanum* ‘Hanyuan’ leaf remained consistently high throughout the development stages, and showed two peaks at 86 d AF and 100 d AF, with similar increases in the branch. This trend may be attributed to the application of Mancozeb in the orchard, which acts effectively as foliar fertilization, thereby increasing the content of respective plant elements (Kowalenko and Ihnat, 2010). Mancozeb has a short half-life of 1−3 d, and residues become undetectable within 7 d after spraying (Devi et al., 2014). When the mancozeb application was stopped, the Mn content in *Z. bungeanum* ‘Hanyuan’ leaf and branch declined rapidly, implementing the Chinese prickly ash’s ability to manage excess Mn through transport or metabolic pathways to prevent harm. This fungicide does not negatively impact Chinese prickly ash growth under normal conditions (Skórka et al., 2023). However, based on the trend of Mn changes in *Z. bungeanum* ‘Hanyuan’, it takes 10−14 d for the plant to metabolize excess Mn. Therefore, Mancozeb should be stopped at least half a month before harvest to ensure the balance of elements in the *Z. bungeanum* ‘Hanyuan’ and food safety.

## Conclusion

The leaf weight of *Z. bungeanum* ‘Hanyuan’ increased the most rapidly at 1−39 d AF, followed by slower growth. In the leaves, the changes in N and P content showed a continuous decrease. The changes in K and Mg content remained relatively stable. The changes in Ca, Fe, Cu, and Zn content showed a pattern of ‘decreasing-increasing’. In the branches, the changes in N, P, K, and Mg content showed a continuous decrease. The changes in Ca content showed a pattern of ‘decreasing-increasing’, and the Fe, Cu, and Zn fluctuated. Overall, the nutrient accumulation in the branches and leaves of *Z. bungeanum* ‘Hanyuan’ showed a continuous increase, despite some elements declining during specific periods. The N, P, K, Ca, Mg, Fe, Mn, Cu, and Zn absorption of current-year organs (including branches, leaves, and fruits) were 88.15 kg/ha, 11.05 kg/ha, 103.87 kg/ha, 129.37 kg/ha, 17.25 kg/ha, 1.32 kg/ha, 0.93 kg/ha, 64.57 g/ha, and 272.03 g/ha, respectively, at the fruit mature stage, providing a basis on the nutrient accumulation of the current-year organs of *Z. bungeanum*, fertilizer can be applied to the Chinese prickly ash orchards regularly and quantitatively. The various nutrients showed a decreasing trend during early growth stages, emphasizing the necessity for fertilization. The appropriate periods for nutrient diagnosis were approximately 39−86 d AF for the leaf and 64−100 d AF for the branch. This study provides data support for fertilization management at different stages of the Chinese prickly ash orchard, contributing to the promotion of low-fertilizer, high-efficiency green production in the industry, and facilitating the sustainable development of Chinese prickly ash orchards.

## Data Availability

The original contributions presented in the study are included in the article/supplementary material, further inquiries can be directed to the corresponding author/s.
